# Implementation of health and health-related sustainable development goals: progress, challenges and opportunities – a systematic literature review

**DOI:** 10.1136/bmjgh-2019-002273

**Published:** 2020-08-26

**Authors:** Wafa Aftab, Fahad Javaid Siddiqui, Hana Tasic, Shagufta Perveen, Sameen Siddiqi, Zulfiqar Ahmed Bhutta

**Affiliations:** 1 Department of Community Health Sciences, Aga Khan University, Karachi, Sindh, Pakistan; 2 Centre for Global Child Health, Hospital for Sick Children, Toronto, Ontario, Canada; 3 The Academia, Health Services and Systems Research, Duke-NUS Medical School, Singapore; 4 Center of Excellence in Women and Child Health, Aga Khan University, Karachi, Sindh, Pakistan

**Keywords:** health policy, systematic review, public health

## Abstract

**Introduction:**

While health is one of the Sustainable Development Goals (SDGs), many other ‘health-related’ goals comprise determinants of health. Integrated implementation across SDGs is needed for the achievement of Agenda 2030. While existing literature is rich in normative recommendations about potentially useful approaches, evidence of implementation strategies being adopted by countries is limited.

**Methods:**

We conducted a systematic review with qualitative synthesis of findings using peer reviewed and grey literature from key databases. We included publications examining implementation of health and health-related SDGs (HHSDGs) at national or subnational level published between June 2013 and July 2019.

**Results:**

Of the 32 included publications, 24 provided information at the national level while eight provided information for multiple countries or regions. Our findings indicate that high-level political commitment is evident in most countries and HHSDGs are being aligned with existing national development strategies and plans. A multisectoral, integrated approach is being adopted in institutional setups but evidence on effectiveness of these approaches is limited. Funding constraints are a major challenge for many countries. HHSDGs are generally being financed from within existing funded plans and, in some instances, through SDG-specific budgeting and tracking; additional funding is being mobilised by increasing domestic taxation and subsidisation, and by collaborating with development partners and private sector. Equity is being promoted by improving health service access through universal health coverage and social insurance schemes, especially for disadvantaged populations. Governments are collaborating with development partners and UN agencies for support in planning, institutional development and capacity building. However, evidence on equity promotion, capacity building initiatives and implementation approaches at subnational level is limited. Lack of coordination among various levels of government emerges as a key challenge.

**Conclusion:**

strengthening implementation of multisectoral work, capacity building, financial sustainability and data availability are key considerations to accelerate implementation of HHSDGs.

**Key questions**

**What is already known?**

- Normative literature on Sustainable Development Goals (SDGs) highlights the need for early recognition of interrelatedness of goals, efficient resource allocation, strong government ownership and community engagement as well as institutional coherence, collaboration and coordinated multisectoral approaches.
- Effective governance and appropriate institutions based on values of transparency, accountability, equity and civic engagement are necessary for achievement of SDGs.

**What are the new findings?**

- Health and health-related SDGs are being aligned with existing national development agendas and there is a clear trend towards implementation through existing or new multisectoral and multistakeholder institutions.
- Key challenges to implementation at national and subnational levels include: lack of coordination between different levels of government and with other stakeholders, limited financial resources, high donor dependence, inadequate mainstreaming of SDGs in subnational planning and budgeting, and lack of disaggregated and reliable data.
- Innovative approaches are being adopted, for example, in enhancing funding for SDGs (SDG-specific budgeting and tracking, increasing taxation or subsidies, and collaboration with development partners and private sector) and data generation and collection (population surveys, using routine administrative data, more efficient use of available data from alternate sources such as civil society organisations, think tanks and private sector).

**Key questions**

**What do the new findings imply?**

- Policy makers and implementers should pay more attention to appropriate integrated institutional forms and capacities at national and subnational levels, which support multisectoral and multistakeholder work.
- Sharing of experiences and models of implementation across countries and at regional levels could allow countries to make informed decisions about potentially useful implementation approaches which may work in their context.
- Future research should focus on identifying effective approaches for integrated implementation, impact assessment of health-related goals and effective approaches for monitoring and reporting.

## Introduction

Agenda 2030, comprising of 17 Sustainable Development Goals (SDGs), is far more comprehensive and ambitious than the millennium development goals (MDGs) that concluded in 2015.

While health itself is only one of the 17 goals, other goals include a range of determinants of health, which although not directly related to goal 3, are no less important. Because SDGs are so interconnected,^1 2^ ensuring progress requires integrated implementation so that results can be achieved for many targets and trade-offs might be averted.^3^

While MDGs led to development gains in many countries in multiple areas including health, the world as a whole lagged behind in fulfilling the health goals. According to a review of MDG implementation by the UN Board for Coordination, the experience of MDGs holds key lessons for future development endeavours such as: recognition of interrelatedness of goals; efficient resource allocation; ownership by government and community engagement; collaboration across institutional levels and coordination across sectors.^4^

Addressing key social, economic and environmental dimensions of health and health-related SDGs (HHSDGs) requires a strong focus on governance and implementation.^5 6^ Success in achieving this ambitious agenda will also require much more integrated action across all levels of government and with non-governmental actors.^6^ Therefore, SDG implementation must be monitored and assessed from the early phases of planning and implementation.

Yet little information is available about what implementation strategies countries, particularly low-income and middle-income countries (LMICs), have adopted in the first few years of the SDG era for achieving HHSDGs. Early analysis of such information is necessary to inform policy and practice and to adjust implementation strategies and processes. Therefore, we conducted a systematic review of existing evidence to assess whether: (1) countries are making steady progress on local adaptation (or localisation) of HHSDGs; (2) what strategies for integrated implementation are being adopted and what early lessons are emerging at national and subnational level and (3) what gaps and challenges are evident in HHSDG implementation.

## Methods

### Study design

We used a scoping review methodology, as appropriate for our question,^7 8^ with some modifications to accommodate logistic limitations. These included not considering non-English language publications and limiting non-peer-reviewed literature to the most potentially useful sources.

### Search strategy and information sources

We searched Medline, Embase, CAB Abstracts, CINAHL, Cochrane (CENTRAL Register of Controlled Trials and Database of Systematic Reviews), 3ie Databases of Impact Evaluations and WHO regional databases (WHOLIS). A comprehensive list of grey literature sources was compiled (detailed search strategy in online supplement 1), and after consultation with experts in the field the sources most likely to contribute efficiently towards the literature were selected. These included ‘Google’, ‘Open Gray’, ‘UN high level political forum for SDGs’, ‘UNDP’, ‘UNFPA’, ‘UNICEF’, ‘World Bank’ and ‘IDS bulletin’. Population, Intervention, Comparison and Outcomes elements were reflected in the terms searched, though we did not have C and O components. P was ‘health and related sustainable goals’ whereas I was ‘multisectoral implementation strategies’. We developed search strategy around following terms with some amendments as required by each database: ‘Sustainable Development Goal’, ‘Multisectoral’, ‘Collaboration’, ‘Implementation’, ‘Policy’, ‘legislation’, ‘whole-of-government’, ‘Integration’, ‘Plan of action’. Only English language publications were retrieved.

10.1136/bmjgh-2019-002273.supp1 Supplementary data

### Study framework of analysis

**Figure 1:**
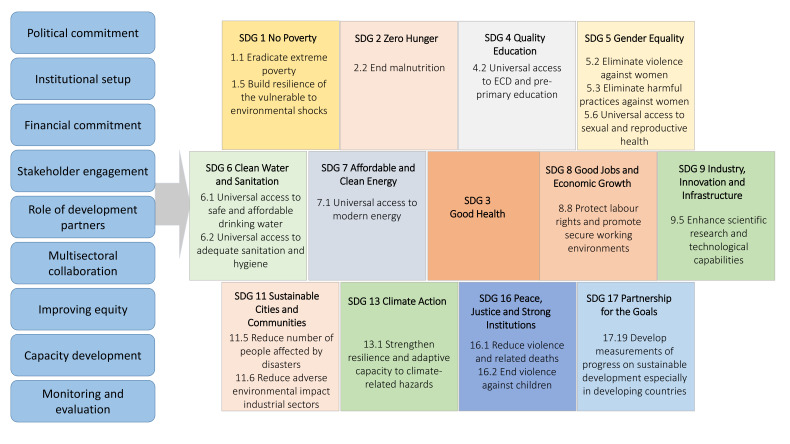
Analytical framework for the review. ECD, Early Childhood Development; MOH, Ministry of Health; HSDGs, health-related Sustainable Development Goals.

In the absence of pre-existing frameworks which could encompass various political and institutional dimensions of HHSDG implementation, we developed a framework of analysis to guide the screening, analysis and synthesis of literature figure 1.

The framework aligns the various stages of policy implementation processes from generating political commitment to monitoring impact and draws on existing frameworks of heath-in-all-policies.^9 10^ The framework comprises nine domains which represent key processes involved in planning and implementation of HHSDGs at national level. Conceptually, the nine domains represent political, technical and institutional conditions that may determine whether and to what extent HHSDG targets and indicators are achieved.

The framework is based on the premise that while high-level political commitment is imperative, to be converted into impact it has to be translated through appropriate institutional setups, adequately funded programmes, meaningful stakeholder engagement^10^ and collaboration across multiple sectors to create impact on health and health-related targets. Monitoring the effectiveness of these processes as well as their impact is also a key consideration. The framework also takes into account defining values for public health policies such as equity.^10^ Health-related SDGs and selected targets in the framework are based on WHO’s 2018 Global Reference List of 100 Core HHSDG Indicators.^11^ This includes SDG3 and its targets in full whereas selected targets and indicators from 12 other SDGs are included.

#### Eligibility criteria

We considered a publication to be eligible if it discussed the implementation of HHSDGs at national or subnational level. We did not apply any restriction by study design. We included literature published between June 2013 and July 2019. Although the SDGs were ratified in 2015, we included the period before 2015 to capture early publications before SDGs were formally adopted.

#### Exclusion criteria

We excluded any publication that provided information solely about multicountry/regional efforts without any national information. We also excluded publications that only provided normative guidance or potential approaches for implementation.

#### Screening and data extraction

Three experienced reviewers independently screened the retrieved records using title and abstract (peer-reviewed articles) or title and snippet (grey literature). Discordant decisions were resolved with consensus after discussion. We followed the same methodology for data extraction for which a predesigned and tested form, based on the analytical framework, was used.

As included publications were not hypothesis testing studies, risk of bias assessment was not applicable.

### Data synthesis and analysis

Initially, we conducted a content analysis of all relevant publications considering each document as a ‘case’. Each publication was read and coded for relevant content using the framework of analysis (figure 1). From each publication, data were extracted for each of the nine domains of the framework in relation to the included HHSDG targets and then synthesised in relevant categories or themes. Particularly useful examples of implementation modalities are included in the explanation of each domain. Challenges to implementation were extracted and synthesised under common themes and are presented in accordance with the domains of the analytical framework (table 1).

**Table 1:** Key challenges to implementation of health and related SDGs

Political commitment	Unstable political environment.^17 21^ Policy and planning incoherence and lack of prioritisation.^12^
Institutional setup	Lack of institutional mechanisms for coordination between national and subnational agencies.^17 22 23 27^
Financial commitment	Limited financial resources.^12–19 21–24 27^ High dependence on donor funding/external assistance.^15 16 19 21 24 27^ Inadequate mainstreaming of SDGs in subnational planning and/or budgeting.^15 17 22 23^ Deleterious effect of climate change on economic productivity and human capacity.^16^ High indebtedness to international financial institutions.^21^
Multisectoral collaboration	Inadequate coordination between national and subnational agencies for multisectoral work.^17 22 23 27^ Inadequate empowerment of local governments.^12^
Stakeholder engagement	Lack of clear roles for various stakeholders.^16 19 22 24 27^ Lack of meaningful involvement of stakeholders/lack of coordination with government.^16 22 27^ Limited involvement of civil society and community,^15 19 22–24 27^ research institutions^14–16 19 24^ and private sector.^15 16 19 24 27^ Lack of resources to maintain well-structured collaborations, fair representation and managing high expectations.^20^
Role of development partners	Poor coordination between development partners.^17^ Development partner priorities take precedence over government.^17^
Improving equity	Appropriately disaggregated data to monitor access and impact across marginalised and disadvantaged groups.^15 23^
Capacity development	Capacity gaps in SDG costing and budgeting, gender mainstreaming, monitoring evaluation, policy formulation, technical capacity and management of statistical information.^12 13 15 17 19 20 23 27^
Monitoring and evaluation	Poor baseline data.^15 27^ Inadequate data management infrastructure and capacity.^15 27^ Focus on data gathering and management but limited analysis and use.^19 27^ Missing private sector data in national data management systems.^27^ Lack of availability of periodic data to monitor progress frequently.^18 19 22 37^ Data reliability issues.^12 19^ Lack of disaggregated data.^12 15 17 21–23 35^ Disharmony between national and subnational targets.^15^ Inadequate funding for data and monitoring.^16^ Exclusive focus on population survey data and inadequate use of routine administrative data.^12^ Limited technical capacity and infrastructure for data collection and management.^20 27^ Heavy reliance on donors for data collection.^12^

Institutional set up.

SDGs, Sustainable Development Goals.

Though the topic and design do not fully lend themselves to the Preferred Reporting Items for Systematic Reviews and Meta-Analyses checklist items, we have used it as a reporting tool to be comprehensive and transparent (online supplement 2).

## Results

**Figure 2:**
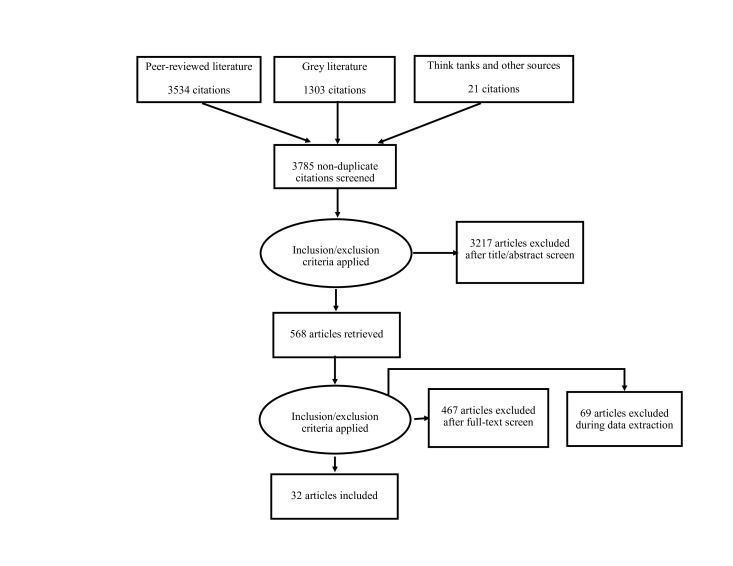
Flow diagram.

Of the total 4858 publication found in the search (3534 peer-reviewed articles and 1324 documents from grey literature), 3785 abstracts were screened after removing duplicates. A total of 3217 publications were excluded by two independent reviewers based on the exclusion criteria described above. Of the total 568 full-text publications assessed, 32 were included in the final analysis (figure 2).

### Characteristics of included publications

**Figure 3:**
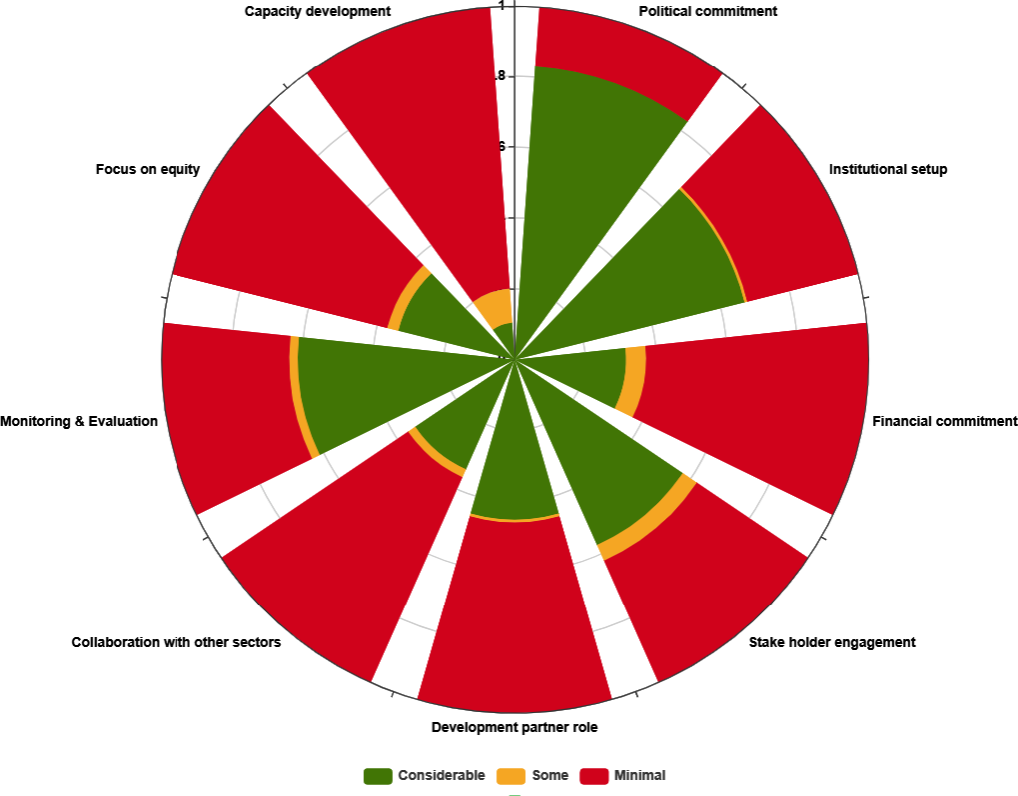
Availability of information about HHSDG implementation by domain for all included countries. HHSDG, health and health-related sustainable development goal.

Twenty-four included studies have findings from individual countries. Of these 13 are from South Asia, nine from Africa and one each from Europe and Latin America and the Caribbean. Five publications presented collective findings from multiple countries in a region (reported separately for individual country); three are from Latin America and the Caribbean and one each from South Asia and Eastern Africa. Three publications provide information about multiple countries across the globe. Detailed characteristics and key findings of each publication are presented in online supplement 3. Figure 3 provides an assessment of availability of information across the nine domains across all countries included in the study. Given the qualitative nature of the results, we did not develop a numerical score to organise the information into three categories, but the two authors who extracted data reached a consensus to categorise any paper as having considerable, some or minimal information. The figure, therefore, presents the amount of information available for the nine dimensions across all countries for which we found any information.

### Implementation modalities of HHSDGs

The findings related to HHSDG implementation modalities are presented by each domain of the framework. In addition to HHSDG-specific findings, we have also included evidence of some implementation strategies that apply to SDGs generally but are still pertinent to HHSDGs.

### Political commitment

Political commitment to SDGs is mostly being framed within the context of broader, often pre-existing, national development aspirations. Governments are leveraging SDGs to achieve improvement in socioeconomic status and meet national development goals^12–18^ and to fulfil regional development commitments.^12 16 17 19^ Most countries, especially LMICs, have chosen to focus on key SDGs based on national priorities and available capacity and resources, which mostly include health but not all health-related goals. For instance, among countries that presented voluntary national reviews at the UN high level political forum on SDGs in 2017, 30% reported on all 17 goals while the rest only reviewed progress for priority goals.^20^

Key challenges to implementation related to political commitment, as well as other domains, are presented in table 1.

### Institutional setup

SDG implementation is being spearheaded by high level political entities lead by heads of government, heads of state and key ministers^13 14 16 17 21–23^ indicating high political commitment. As an acknowledgement of the interrelated nature of SDGs, implementation is often being overseen or led by multiagency structures such as planning commissions,^14–16 19 22–24^ cabinet committees,^22^ interministerial forums,^14^ and parliamentary committees.^16 21^ While most countries are relying on pre-existing entities for implementation, new structures have also been set up given the need for collaborative governance required across government and civil society. For instance, Brazil’s government has created a National Commission for SDGs in the Office of the President with representation of relevant central ministries, state and district governments, civil society representatives, municipal governments and the national institutes for statistics and economic research.^25^ According to a 2018 United Nations Department of Economic and Social Affairs report, in 60 countries studied across the world, 27 have created new cross-sectoral entities for SDG implementation creating integration at horizontal (different sectors and institutions) and vertical (national and subnational government) levels.^26^

Institutional structures for subnational implementation have been clearly developed in some countries,^14 17 19 22–24^ most often in decentralised governance systems, but remain unclear in others.

### Financial commitment

In most countries financial allocation is ensured by incorporating SGDs into currently funded development strategies and plans.^12–17 19 21–24 27 28^ Some countries have reoriented budgeting in ways that SDG expenditures are traceable to allow assessment of financial allocation. For instance, SDG-specific outlays in line ministry budgets in Afghanistan, SDG coding in budgets to track SDG-related expenditures in Nepal,^15^ and cross-matching of budgets and SDG priorities to estimate SDG-specific funds in Mexico.^25^ Budget estimations have been done for additional financial resources needed to achieve SDGs.^12 14 20 22 25^ For instance, Bangladesh has estimated that an additional US$928.48 billion will be needed to fully implement SDGs in the country.^14^

Various strategies that are being adopted to increase funds and to use them more efficiently include: (1) leveraging funds from private sector^13 17 19 25^ and development agencies,^13 15–17 24 27^ increasing fiscal space in general by increasing general tax revenue,^12^ and for health by instituting ear-marked taxes, for example, AIDS levy and mobile communication taxes for health in Zimbabwe;^21^ (2) enhancing accountability in financing, for example, result-based or performance-based financing where future funding to programmes is tied to improvement in indicators;^12 17 21 24 25^ (3) using budgeting strategies to focus on priority goals and to take advantage of synergies between different goals by: prioritising key sectors such as health, nutrition, and education for allocation, and by protecting their funding from budgetary fluctuations;^13 25^ and by restructuring budgeting processes to focus on priority goals, for example, disaggregating spending by gender in all sectors to support fiscal policies that value women’s contribution to the economy in Mexico.^25^

### Multisectoral collaboration

Of the potential mechanisms which can be used for multisectoral collaboration for health,^29^ the most commonly used we found are: cabinet/interministerial committees and secretariats,^12–14 17 19 22 24^ interdepartmental committees and units,^13 17 24^ and parliamentary committees.^16 21^ Examples include the interministerial SDG monitoring and implementation committee of secretaries of 21 ministries in Bangladesh;^14^ ministries of finance and/or planning working bilaterally with other ministries in Denmark and Tanzania;^12 20^ thematic clusters of ministries or departments with related portfolios in Pakistan,^22^ Rwanda, Uganda, Zimbabawe and Zambia;^30^ and mutlisectoral setups for noncommunicable disease control in Iran^31^ and India.^32^ A few countries have taken more comprehesive approaches such as heath-in-all-policies^20 26^ and social-determinants-of-health approach.^12^

While most coutries have reported on structures, far less information is available on the processes being used to implement multisectoral collaboration. Of the various process-based approaches for multisectoral work described by Boston and Gill^33^ some evidence is available for information sharing^12 14 17 27^ and aligning sectoral activities,^12 14 27^ but less so for resource sharing,^12 14^ shared responsibilities or accountability. Similarly, some countries have reported on structures for multi-sectoral collaboration at local levels^14 19 24^ but information about processes to implement local-level collaboration is scarce.

### Stakeholder engagement

The most commonly involved stakeholders in SDG implementation are private sector,^12 15 16 19 23 27^ think tanks and academia,^12 14 15 19 22 27 34^ development partners^15–17 19 21 22 27^ and civil society organisations.^12 14–19 21 24 27 35 36^ The role of civil society organisations is particularly important in bringing together key population groups and highlighting their issues, for example, women, youth and the poor. For instance, the Asocia 2030 project in Chile has more than 350 civil society organisations working on gender equality, poverty, hunger, ill health and building resilient infrastructure.^25^ Some governments are taking a whole-of-society approach to stakeholder engagement by formally involving civil society, private sector and development partners in SDG planning and implementation structures of the government.^12 14 17^

National and regional think tanks and multilateral agencies are facilitating experience sharing between regional countries in Southeast Africa,^30^ South Asia^37^ and Latin America.^25 38^

The various roles being played by stakeholders in SDG implementation include: devising mechanisms for determining goals and targets and implementation and monitoring mechanisms;^12 14 18 20 24^ monitoring the implementation of SDGs^14 17 20 24^ such as by providing inputs in national voluntary reviews or creating independent monitoring reports;^20^ providing technical expertise;^12 14 22 23^ bringing attention to equity issues;^12 14 15 17 19 23^ providing financial support^13 15–17 19 24 25 27^ and raising awareness about SDGs.^12 17 19 21 27^

### Role of development partners

The role and presence of development partners and donors varies by focus area including policy guidance, financing, research and advocacy. Bilateral and multilateral agencies are actively supporting in health and related areas. Regional development banks are actively involved in South Asia^37^ and Africa.^30^ Key roles being played by development partners include setting up implementation structures and financing them,^13 22 39^ supporting coordination mechanisms, monitoring and funding^21 27^ and SDG promotion and dissemination activities.^18 21 39^

The most prominent role in supporting the implementation of^39^Agenda 2030 is being played by UN agencies.^39 40^ United Nations Development Programme is working in multiple countries in establishing institutional structures,^22 39^ SDG financing and promotion,^17 21 39^ advocacy and awareness raising,^16 17 21 39^ budgetary estimation,^25^ aligning SDGs with existing national policies and strategies^25 39^ and sensitising legislators and enhancing policy makers’ capacity.^21 39^

### Improving equity

In terms of equity, countries are making efforts to specifically focus on the needs of disadvantaged populations. Some groups that are being considered are: women, children, elderly, people with disabilities, sexual minorities, indigenous peoples and migrants.^20^ Socioeconomic inequalities and urban-rural differences are also being addressed for instance through social protection policies and reviewing resource allocation mechanisms.^12 17^ For instance, Kenya has reviewed its resource allocation formula to prioritise poorer population and has set up an Equalisation Fund to allocate more public resources to high poverty areas.^17^ In health service access equity is being ensured through promoting universal health coverage^14–17 21 27^ by instituting basic and essential packages of health services^14 27^ and financing strategies such as social health insurance.^17^

### Capacity development

While limited evidence is available about capacity development initiatives, more information is available about identification of capacity gaps. Key identified needs are in the areas of SDG costing and budgeting, gender mainstreaming, monitoring and evaluation,^15 23 27^ policy formulation and technical capacity and management of statistical information, particularly administrative data.^12 13 17 19 20^ Some examples of SDG-focused capacity building include: programmes for knowledge and skill building of parliamentarians to enhance legislative^21 25^ capacities of subnational governments to align strategies and policies with SDGs^21^ and enhacing capacity of health sector workers.^16 17 34 41 42^

### Monitoring and evaluation

In most countries work is underway in defining priority goals, targets and indicators. Assessment and strengthening of statistical system capacity to ensure availability of data for monitoring progress on chosen indicators is also underway. For instance, Thailand has conducted national burden of disease study and health estimates^43^ and has devised a comprehensive methodology to assess progress towards universal health coverage using data from household surveys, facilities, disease registries and research.^44^ Where data for monitoring are not available, various approaches being used to enhance data availability are: using data from global databases until country systems are more mature;^45^ reconsidering the periodicity of national surveys to allow frequent availability of data;^16^ using data from alternate sources such as think tanks and civil society organisations^12 17^ and using technology to make data collection more efficient, for example, satellite technology for household surveys.^20^

Since national aggregates can hide deep subnational inequities, subnational data disaggregation is recognised as a priority but also a challenge in many places. Developed countries often have more developed data systems and capacity and some have offered support to LMICs for help in developing their statistical systems and capacity.^20^ Performance contracting is being used in some places to enhance accountability in SDG target attainment.^12 17 24^

## Discussion

The findings from existing literature reveal key institutional mechanisms and strategies that countries are taking for implementing SDGs, as well as the commonly faced challenges and potential solutions. Our study is one of the first to systematically synthesise literature on implementation modalities of health and related SDGs at national and subnational level.

Existing literature on sustainable development and health is predominantly normative and highlights the fundamental role of effective governance and appropriate institutions based on values of transparency, accountability, equity and civic engagement.^46 47^ Our findings suggest that current implementation efforts recognise the role of political will and accountability as the HHSDG agenda often has the oversight of parliaments. However, clear mechanisms and measures of accountability are not currently documented in implementation literature on the subject. While participatory decision making is often stated as a guiding principle for policies, our findings suggest that substantive and systematic participation of civil society and other stakeholders is inadequate and needs more attention. The discrepancy could be resolved by clear articulation of the roles of various stakeholders leveraging on their strengths. For instance, academia and think tanks could play a vital role in knowledge creation, policy guidance and bridging the gaps between public concerns and policy agendas. While literature, particularly from South Asia and Eastern Africa, reflects involvement of think tanks in localisation, concerns have been raised that their strengths are underused, and engagement efforts could be more organised and outcome-oriented. These values also need to be reflected in subnational governance systems, of which existing evidence is limited.

Although appropriate institutional arrangements are subject to political, social and cultural context, there is a consensus in current literature on the need for institutional forms to reflect the universality of the notion of sustainable development and the necessity of policy coherence across sectors.^26 48 49^ Our findings show that multisectoral structures with health at the centre, or as a crucial component, are evolving. How effective these structures are and whether and under what conditions can health sector successfully lead the multisectoral agenda need further investigation. Moreover, there is little evidence on the appropriate framing and advocacy approaches which the health sector could adopt to convince diverse sectors to contribute to the health-in-all-policies agenda.

A key implementation concern about the sustainable development agenda has been the inadequacy of available government funding and this has emerged as the most prominent challenge in our findings. Development partners and private sector (e.g., corporate and philanthropic sectors) are being engaged to enhance funding, yet crucial gaps remain in domestic funding in many countries. As recommended in the Addis Ababa Action Agenda on development financing,^50^ it is desirable to rely on domestic resources for increasing funding. While measures are being adopted to raise domestic funding and align the resources of non-government partners with the SDG agenda, further evidence is needed on innovative ways to achieve targets such as target 3.8 on universal health coverage, especially in low-resource settings.

Finally, the most prominent feature of SDG planning in most countries is the focus on choosing indicators and collecting required data. The key messages emerging from our review suggest that a multipronged approach may be necessary by: (1) taking advantage of a large amount of routine administrative data rather than just relying on population-level surveys, which are more resource-intensive and therefore only possible after intervals of multiple years; (2) making more efficient use of available data from alternate sources such as civil society organisations, think tanks and private sector; (3) strengthening capacity for reliable production, analysis and utilisation of data at all levels; and (4) making sure disaggregated data are available by various population characteristics, for example, race, ethnicity, gender, area of residenceand socioeconomic status depending on what is appropriate for each country, but keeping the focus on inequities and disadvantaged populations thus making sure that ‘no one is left behind’. A key consideration in monitoring integrated implementation should be that HHSDGs are not just a collection of targets and indicators but a set of interacting components with multiple potential synergies and trade-offs. These interactions may require much more integrated systems of monitoring and impact assessment.^51^

A limitations of the study is that it looks only at those implementation approaches that are documented in literature. Even though we undertook a detailed study of grey literature, it is likely that other implementation approaches are being used but not captured here if they are not documented. Our use of English language literature means that information from some regions may be limited. Moreover, documents in the review are predominantly from LMICs and mainly from South Asia, Eastern Africa and some from Latin America and the Caribbean, which implies that the findings should be interpreted in view of this representation.

### Implications for future research

Key areas that need further research to guide SDG implementation include: effective implementation strategies working at the intersection of governance, accountability and multisectorality, cost-effective means of integrated implementation, ways of measuring health impact of non-health sector work, and high-quality monitoring approaches and data for accountability and coordinated achievement of goals.

## Conclusion

The study suggests that implementation efforts for HHSDG implementation are at various stages in various countries. More attention is needed to strengthen implementation of multisectoral work, capacity building, financial sustainability and data availability. Areas for future research include pathways for integrated implementation, impact assessment of health-related goals and effective monitoring approaches.
