# Supplementary material for: Implementation of health and health-related sustainable development goals: progress, challenges and opportunities – a systematic literature review
Source: BMJ Glob Health. 2020 Aug 26;5(8):e002273. doi: 10.1136/bmjgh-2019-002273 (PMC7451474; doi:10.1136/bmjgh-2019-002273)
Supplement: Supplementary data [file bmjgh-2019-002273supp001.pdf]

Supplement 1 - Search strategy for MEDLINE database

MEDLINE

1. Sustainable Development Goal*.mp.

2. SDG*.mp.

3. 1 or 2

4. exp PHENOTYPE/ or phenotype*.mp. or subdural grid*.mp. or glycemia standard deviation.mp. or peptide*.mp. or exp Amino Aci ds/ or amino acid*.mp. or diglucoside*.mp. or histone*.mp.

5. 3 not 4

6. exp Intersectoral collaboration/ or Intersectoral.mp. or Multisecotral.mp. or Cooperation.mp. or Collaboration.mp. or Impl ementation.mp. or Policy.mp. or legislation.mp. or rules.mp. or Regional.mp. or global.mp. or "whole-of-government".mp. or "Joined-up government".mp. or "Healthy public policy".mp. or "Health-in-all-policy".mp. or HiAP.mp. or "Health-in-all-policies".mp. or Integrat*.mp. [mp=title, abstract, original title, name of substance word, subject heading word, floating sub-heading word, keyword heading word, organism supplementary concept word, protocol supplementary concept word, rare disease supplementary concept word, unique identifier, synonyms]

7. 5 and 6

8. limit 7 to ed=20130601-20190731

Embase:

1. Sustainable Development Goal*.mp.

2. SDG*.mp.

3. 1 or 2

4. exp PHENOTYPE/ or phenotype*.mp. or subdural grid*.mp. or glycemia standard deviation.mp. or peptide*.mp. or exp Amino Aci ds/ or amino acid*.mp. or diglucoside*.mp. or histone*.mp.

5. 3 not 4

6. exp Intersectoral collaboration/ or Intersectoral.mp. or Multisecotral.mp. or Cooperation.mp. or Collaboration.mp. or Implementation.mp. or Policy.mp. or legislation.mp. or rules.mp. or Regional.mp. or global.mp. or "whole-of-government".mp. or "Joined-up government".mp. or "Healthy public policy".mp. or "Health-in-all-policy".mp. or HiAP.mp. or "Health-in-all-policies".mp. or Integrat*.mp. [mp=title, abstract, heading word, drug trade name, original title, device manufacturer, drug manufacturer, device trade name, keyword, floating subheading word, candidate term word]

7. 5 and 6

8. limit 7 to ed=20130601-20190731

CAB Abstracts

1. Sustainable Development Goal*.mp. [mp=title, abstract, heading word, drug trade name, original title, device manufacturer, drug manufacturer, device trade name, keyword, floating subheading word, candidate term word]

2. SDG*.mp.

3. 1 and 2

4. exp PHENOTYPE/ or phenotype*.mp. or subdural grid*.mp. or glycemia standard deviation.mp. or peptide*. mp. or exp Amino Acids/ or amino acid*.mp. or diglucoside*.mp. or histone*.mp.

5. 3 not 4

6. exp Intersectoral collaboration/ or Intersectoral.mp. or Multisecotral.mp. or Cooperation.mp. or Collaboration.mp. or Implementation.mp. or Policy.mp. or legislation.mp. or rules.mp. or Regional.mp. or global.mp. or "whole-of-government".mp. or "Joined-up government".mp. or "Healthy public policy".mp. or "Health-in-all-policy".mp. or HiAP.mp. or "Health-in-all-policies".mp. or Integrat*.mp. [mp=title, abstract, heading word, drug trade name, original title, device manufacturer, drug manufacturer, device trade name, keyword, floating subheading word, candidate term word]

7. 5 and 6

8. limit 7 to (abstracts and full text & cab abstracts fulltext and last 6 years)

Supplement 2 - PRISMA checklist 2009

Section/topic	#	Checklist item	Reported on page #
**TITLE**			
Title	1	Identify the report as a systematic review, meta-analysis, or both.	Yes. Title page.
**ABSTRACT**			
Structured summary	2	Provide a structured summary including, as applicable: background; objectives; data sources; study eligibility criteria, participants, and interventions; study appraisal and synthesis methods; results; limitations; conclusions and implications of key findings; systematic review registration number.	Yes.<br>Registration number not applicable.
**INTRODUCTION**			
Rationale	3	Describe the rationale for the review in the context of what is already known.	Yes.
Objectives	4	Provide an explicit statement of questions being addressed with reference to participants, interventions, comparisons, outcomes, and study design (PICOS).	Yes. Explained in introduction and methods.
**METHODS**			
Protocol and registration	5	Indicate if a review protocol exists, if and where it can be accessed (e.g., Web address), and, if available, provide registration information including registration number.	NA. No protocol registered.
Eligibility criteria	6	Specify study characteristics (e.g., PICOS, length of follow-up) and report characteristics (e.g., years considered, language, publication status) used as criteria for eligibility, giving rationale.	Yes.
Information sources	7	Describe all information sources (e.g., databases with dates of coverage, contact with study authors to identify additional studies) in the search and date last searched.	Yes.

Search	8	Present full electronic search strategy for at least one database, including any limits used, such that it could be repeated.	Detailed search strategy Supplement 1. Key information in methods section.
Study selection	9	State the process for selecting studies (i.e., screening, eligibility, included in systematic review, and, if applicable, included in the meta-analysis).	Yes.
Data collection process	10	Describe method of data extraction from reports (e.g., piloted forms, independently, in duplicate) and any processes for obtaining and confirming data from investigators.	Yes.
Data items	11	List and define all variables for which data were sought (e.g., PICOS, funding sources) and any assumptions and simplifications made.	Yes.
Risk of bias in individual studies	12	Describe methods used for assessing risk of bias of individual studies (including specification of whether this was done at the study or outcome level), and how this information is to be used in any data synthesis.	NA.
Summary measures	13	State the principal summary measures (e.g., risk ratio, difference in means).	NA.
Synthesis of results	14	Describe the methods of handling data and combining results of studies, if done, including measures of consistency (e.g., I^2) for each meta-analysis.	Qualitative synthesis on the basis of analytical framework explained.

Supplement 3 - Key characteristics and findings of included publications

Author/Organisation, year	Setting	Title	Key findings related to implementation of health and health related sustainable development goals
**Peer reviewed literature**			
Biswas *et al* 2017 (34)	Bangladesh	Shifting paradigm of maternal and perinatal death review system in Bangladesh: A real time approach to address sustainable developmental goal 3 by 2030	Bangladesh scaled-up a system of maternal and perinatal death review nationally in 2016. The system was developed with collaboration between government and experts and implementation was preceded by capacity building of health workers. It captures maternal and perinatal deaths from community and facilities, including from hard-to-reach areas, and integrates the information in District Health Information System 2. The system allows real time monitoring of deaths and their causes, based on verbal autopsy, from national to local levels allowing health managers and policy makers to make evidence-based decisions to reduce maternal and perinatal deaths.<br>Key challenges include maintaining quality and validity of information, appropriate utilization of findings in decision making, and accountability for outcomes.
Peykari *et al* 2017 (31)	Iran	National action plan for non-communicable diseases prevention and control in Iran; a response to emerging epidemic	The establishment of Iran's national Noncommunicable Disease Committee and the process of development of the national action plan has lessons for other countries in setting up multi-sectoral institutions structures for HSDG achievement. The planning process involved high level of political commitment illustrated by the involvement of the President and key health and non-health ministries; high participation from academia and national and sub-national level policy makers and implementers; multi-sectoral collaboration and clearly defined relationships and accountability, strategies and activities, stakeholders and their roles, outcomes, evaluation criteria and needed resources; and target prioritization based on national needs. Key challenges were lack of adequate budget, inadequate engagement of non-governmental actors, inadequate qualified human resources, poor information and communication, poor coordination and resistance of some organizations.
Galli *et al* 2018 (45)	Montenegro	Think globally, act locally: Implementing the sustainable development goals in Montenegro	The localization of SDGs within Montenegro's National Strategy for Sustainable Development (NSSD 2030) involved: participation of national and international experts, various ministries and civil society; identification of six priority areas; a detailed monitoring and reporting framework; assessment of capacity to measure chosen indicators; identification of data sources including local, national and proxy data; identification of areas for legislative change; mapping of discrete and composite indicators. Montenegro was found to be able to track 137 SDG indicators by 2019. Identified needs were: updating plan every 2-3 years, research needs, integrated monitoring framework, cross-sectoral approaches, political support and financing.
Uwizeye *et al* 2018 (41)	Rwanda	Building Nursing and Midwifery Capacity	Lack of trained human health resource is a potential barrier to achievement of SDG 3. Rwanda's Human Resources for Health (HRH) Program is training and building capacity if nurses through an academic collaboration between the

Author/Organisation, year	Setting	Title	Key findings related to implementation of health and health related sustainable development goals
		Through Rwanda's Human Resources for Health Program	ministry of health 25 US-based teaching institutions. The structured program with a faculty twinning process with international academic institutions makes the program sustainable.<br>The HRH program is an example of aid with national ownership and alignment with the government's goals. This could be a model of capacity building for SDG 3 for other low-resource setting through international academic partnerships.
Mondal *et al* 2018 (32)	India	India's NCD strategy in the SDG era: are there early signs of a paradigm shift?	India's strategic approach to tackling non-communicable diseases as part of SDGs shows high political commitment, systematic prioritization of targets with clear responsibilities, consultations with other ministries, states, international organizations and academia. There is systematic mapping and engagement of non-health sectors and envisages a shift in the government's role from priority setting to 'policy broker'. This represents a shift from the siloed MDG approach to a more integrated, multi-sectoral SDG approach. Challenges include increasing civic engagement, accountability and strengthening regulation.
Witthayapipopsakul *et al* 2019 (44)	Thailand	Achieving the targets for universal health coverage: how is Thailand monitoring progress?	Thailand uses four key tools to monitor the three dimensions of universal health coverage: national surveys; administrative and facility data; disease registries; and research. Population coverage for health insurance schemes is measured using civil registration and vital statistics and a household survey; service coverage is measured using surveys, facility and administrative data and disease registry; financial protection is measured using socioeconomic survey as well as research using financial incidence analysis and benefits incidence analysis. Enabling factors were: supportive leadership, well-developed infrastructure, effective collaboration. Challenges are: migrant data tracking, utilizing data for systems improvement.
Bundhamcharoen *et al* 2017 (43)	Thailand	Contributions of national and global health estimates to monitoring health-related Sustainable Development Goals in Thailand	Thailand's national burden of disease study and national health estimates (NHE) system were developed with support from WHO, international academic institutions and development partner funding, utilizing data from vital registration, hospitals, disease notification and household surveys. Capacity building has been institutionalized by research training collaboration between Thai and international universities. Data analysis and presentation is based on policy needs and impact on informing evidence-based policies is evident in use in multiple national policies and strategies, re-orientation of policy priorities towards high-burden diseases. Challenges include lack of institutional capacity to generate and use NHEs, data quality, and effective utilization of data to drive action.
**Gray literature**			
Afghanistan, 2017 (27)	Afghanistan	Policy Research Institutions and Health Sustainable Development Goals: Building Momentum in South Asia. Country Report: Afghanistan	Afghanistan has formulated a three-phase SDGs process (i) nationalization (ii) alignment and (iii) implementation. The Afghan Central Statistics Office is responsible for restructuring national surveys to record national SDG indicators. Health related targets and indicators are modified to fit the national context.<br>Lack of capacity and resources, poor baseline data, a focus on data management rather than programmatic change and transformative governance, a failure to recognize the role of the private sector and lack of in-depth engagement of non-state actors are some of the challenges for SDG implementation

Author/Organisation, year	Setting	Title	Key findings related to implementation of health and health related sustainable development goals
Bangladesh, 2017 (14)	Bangladesh	Policy Research Institutions and the Health SDGs: Building Momentum in South Asia (Bangladesh study)	Prime Minister's Office is leading the national SDG agenda. Health, Population and Nutrition Sector Program 2016-2020 of the Ministry of Health under the 7th five-year plan (2017-2021) focuses on attainment of health and related SDGs. Priority SDGs are 2, 4, 5, 6, 10, 11 & 16. The Access to Information program of the Prime Minister's Office, Planning Commission and Bangladesh Bureau of Statistics designed and developed SDG Tracker to create a data repository for monitoring the implementation of the SDGs with technical support from UNDP and USAID. Identification of data gaps has been done. Mapping of sectoral responsibilities has been done but progress in multi-sectoral engagement is limited. Challenges include: Lack of capacity for evidence-based policy making.
Bhutan, 2017 (19)	Bhutan	Policy research institutions and the health related SDGs: building momentum in South Asia. Country Report, Bhutan.	The Gross National Happiness Commission, the central planning and coordinating agency, incorporates SDGs into the national plan, programs and strategies. Currently, 134 SDG targets out of the 143 relevant SDG targets have been successfully integrated into the 11th Five Year Plan. Ministry of health is the lead agency responsible for the health sector plan in collaboration with relevant agencies and sectors.<br>Major challenges include lack of resources, lack of periodic data, sensitization, capacity building and engagement of stakeholder
Ethiopia, 2017 (16)	Ethiopia	Scoping Study of National Level Institutional Arrangements for Health Related SDGs Implementation and Monitoring in Ethiopia	Ethiopian government has aligned SDGs with its national Growth and Transformation Plan. Priority SDGs are: 1, 2, 3, 5, 9 and 14. Implementation is steered by the National Planning Commission. National and sectoral M&E frameworks are in place. Planning department in each sector is responsible for monitoring. Devolution of power and resources is one of the key characteristics of the Plan. There are no SDG-specific earmarked funds; allocated sectoral budgets for SDGs; development partners provide some funding for health. Major challenges include: defining and aligning stakeholder roles; mobilizing financial resources; strengthening of data infrastructure and availability of disaggregated data; capacity for implementation; climate change induced drought with negative impacts on economy and human capacity; inadequate financial resources; unpredictability of aid, loans and foreign direct investment.
India, 2017 (23)	India	Policy Research Institutions and the Health SDGs in India	The National Institution for Transforming India (NITI) Aayog and the Ministry of Statistics and Program Implementation are leading implementation of SDGs by the Centre the States. SDGs are being implemented into a 15 year Vision, a 7 year Strategy', and a 3 year action agenda.<br>NGOs and community organizations are collaborating with national, state and local government institutions and international organizations for effective implementation of health SDGs. National Health Policy 2017 is aligned with SDGs. Major challenges include weak inter-sectoral convergence for better health outcomes and need for a data bank and dissemination unit for the nodal national body for SDGs.
Kenya 2017 (17)	Kenya	Situational analysis of the preparedness to roll out and domesticate the health and health related SDGs in Kenya	Agenda 2030 timelines overlap with Kenya Vision 2030 which enables the country to shadow the SDGs in terms of National Development Plans and Programs. The Ministry of Devolution and Planning is responsible for the overall management and coordination of implementation, monitoring and reporting. Kenya Integrated Household and Budget Survey 2016 data is the baseline. Preparation for Medium Term Plan III (MTP) involves stakeholders who will be represented in the National MTP Coordination Forum and the Medium Term Sector Working Groups. The

Author/Organisation, year	Setting	Title	Key findings related to implementation of health and health related sustainable development goals
			preparation of the County Integrated Development Plans is also consultative and therefore creating ownership. The Revenue Allocation Formula was reviewed to prioritize the Poverty Index among resource sharing criteria. The Equalization Fund allocates more public resources to counties with high poverty index. Challenges are: Inadequate coordination between national and sub-national governments; high political turnover; data unavailability; lack of engagement of health policy think tanks.
Nepal, 2017 (15)	Nepal	Policy Research Institutions and the Health SDGs: Building Momentum in Nepal. Country report, Nepal	SDG and UHC commitment is articulated in National Health Policy 2014 and the new constitution (2015). Nepal has well-established institutions for SDG implementation at national level but limited at subnational level. Four means of implementation are: finance, technology, institutions & their capacity, and partnership. Nepal Health Sector Strategy (2015-2020) adopted four pillars related to SDG3: equitable access to health services, quality health services, health systems reform and multi-sectoral approach. Stakeholders such as private sector, CSOs, cooperatives and development partners are involved in SDG localization. Sector Wide Approach has been adopted in health and related sectors. Main challenges include: mainstreaming SDGs into subnational planning and budgeting systems; unavailability of disaggregated data; limited financial and technological resources; outcome and impact level data limited.
Pakistan, 2017 (22)	Pakistan	Policy research institutions and the health related SDGs: building momentum in South Asia. Pakistan country study.	SDGs Unit have been established but the pace of work is comparatively slow and needs to be expedited. Need to enhance coordination among NGOs and opportunities for networking for them to be abreast with the developments around SDGs. Capacity building of local government to plan, implement and monitor SDGs is required. Further work is needed to get for indicators where it's missing and to utilize existing data to inform policy. Setting up standard data protocols and developing coordination mechanisms Federal and Provincial Statistical Bureaus is required.
Rwanda, 2017 (24)	Rwanda /	Analysis of the Integration of Health and Health Related SDGs in Rwanda	Senate and parliament are responsible for oversight of SDG implementation, the cabinet for strategic direction and Sector Working Groups (government and other stakeholders) for technical consultation. Ministerial clusters are responsible for addressing inter-sectoral issues. Currently, government is able to fund only 66% of health sector funding needs. Stakeholders as well as citizens are engaged by participation in various forums e.g. Umuganda. Major implementation challenges include: effective engagement of stakeholders, communication with citizens, financial resources, linkages with research institutions in other countries.
Sri Lanka, 2017 (35)	Sri Lanka	Has Sri Lankan Health Policy Research Institutions Played an Active Role in Achieving Health related SDGs?	Ministry of health with support of WHO has worked on planning and monitoring health SDGs by analyzing the compatibility of SDG core health indicators, aligning them with available indicators and planning a monitoring framework on SDG3. NGOs and CSOs are involved in planning and implementing health related activities.
Tanzania, 2017 (12)	Tanzania	Tanzania and the Sustainable Development Goals: Has Tanzania	SDGs are integrated in national policy and plans. However better coordination and collaboration is required, e.g. linking datasets to enable pooling of information. Priority Goals based on national development preferences are: 1, 2, 3, 4, 5, 6, 7, 9 and 17. Implementation will be operationalized through the Local Economic Development Approach.

Author/Organisation, year	Setting	Title	Key findings related to implementation of health and health related sustainable development goals
		prepared to roll-out and domesticate the health SDGs?	The Ministry of Finance and Planning is be responsible for mobilizing government funds and innovative financing; coordinating, and establishing stakeholders; and providing guidelines to local governments.<br>Challenges for implementation: risks posed by corruption, inadequate resource mobilization, policy-planning incoherence and lack of prioritization, inadequate decentralization.
Uganda, 2017 (13)	Uganda	Readiness of Uganda to roll out Health and Health Related Sustainable Development Goals	SDGs have been incorporated in the National Development Plan II. The SDG Coordination Framework is operated through: SDG Policy Coordination Committee, SDG Implementation Steering Committee, SDG National Task Force and SDGs Technical Working Groups. Challenges include: insufficient resources for local government, to lack of capacity, limited community participation. Work is needed on: adequate engagement of Health Research Institutions, investments in public infrastructure, reforming public procurement, rallying the private sector, civil society, citizens and other partners.
Zambia, 2017 (18)	Zambia	Assessing Zambia's Preparedness to implement sustainable development goals on Health	SDGs are being integrated with existing policies and plans such as National Health Policy, Seventh National Development Plan - 2017-2021 and Zambia National Health Strategic Plan 2017-2021. Stakeholder engagement is being done but more efforts are needed. Challenges: lack of dedicated leadership, inadequacies in localization & prioritization of SDGs so as to align the indicators to the Zambian context. There is near-lack of a central monitoring and evaluation system to track progress across sectors. Real-time data acquisition and reporting for SDGs monitoring is not adequate.
Zimbabwe (21)	Zimbabwe	How are countries prepared to roll out and domesticate Health SDGs–Zimbabwe Report	The Ministry of Macroeconomic Planning and Investment Promotion is responsible for coordinating implementation of the SDGs supervised by the Office of the President and Cabinet. There is a Parliamentary Thematic Committee on SDGs .A Steering Committee, chaired by the Chief Secretary and represented by all line ministry Permanent Secretaries and the heads of the UN agencies provides overall guidance and strategic leadership. A Technical Committee comprised of ministries, UN agencies, civil society, private sector and development partners guides technical processes and technical backstopping. Fiscal resources are limited but there is positive participation from private sector.
South Asia, 2017 (37)	Pakistan, Bangladesh, Nepal, Afghanistan, Bhutan, India & Sri Lanka	Policy Research Institutes and the Health-related SDGs: Building Momentum in South Asia	The role of non-government stakeholders in accelerating the growth on SDGs is important and needs support. Policy research institutions have recommended establishing a forum at regional and country level to coordinate and collaborate with each other to work in a collective manner to implement the SDGs. A capacity need assessment is also required if the support of policy research institutions is to be planned. This type of work has been done in accordance with IDRC's think tank initiative (TTI) in the past.<br>Advocacy and training sessions of media are needed to enable them to play an effective role in awareness raising and support government in promoting SDGs. Policy research institutions can play a role in monitoring and accountability.
South East Africa, 2017 (30)	Ethiopia, Kenya, Rwanda,	Status of Implementation for Health-Related Sustainable Development	In all seven countries pre-existing development frameworks with elements of universal health coverage aspirations support the implementation of SDGs. The momentum generated by MDGs is another enabler. Understanding and adoption of SDGs at population-level remains inadequate. Health policy

Author/Organisation, year	Setting	Title	Key findings related to implementation of health and health related sustainable development goals
	Tanzania, Uganda, Zambia, Zimbabwe	Goals (SDGs) in Seven Countries of Eastern and Southern Africa	think tanks are active and have official role in implementation structures in Rwanda and Ethiopia. There is a clear role of information sharing and technical collaboration through think tanks as regional hubs as well as globally.<br>Common issues across the seven countries are: high poverty, rapid urbanization, high population growth, impact of climate change and threats to food security and health, double burden of infectious and non-communicable diseases.
ECLAC, Latin America and the Caribbean, 2017 (25)	Countries in Latin America and the Carribean region of the UN	Annual report on regional progress and challenges in relation to the 2030 agenda for sustainable development in Latin America and the Carribean	The regional countries are struggling with weak recovery of international economic conditions and trade. It is essential to pursue and broaden multilateral international cooperation. Policymaking requires a multi-stakeholder cross-sectoral institutional foundation and technical capacities for proper policy design and evaluation. Without strengthened national statistical systems it will be impossible to establish whether adequate progress is being made towards the Sustainable Development Goals. With such varied and ambitious objectives, the efforts involved in designing, measuring and interpreting a large number of indicators and voluminous data are complex.
ECLAC, Latin America and the Caribbean, 2018 (38)	Countries in Latin America and the Carribean region of the UN	Second annual report on regional progress and challenges in relation to the 2030 Agenda for Sustainable Development in Latin America and the Caribbean	Multi-stakeholder and cross-sectoral institution foundation and the technical capacities for proper policy design and evaluation are mandatory and being developed. Strengthened national statistical systems are imperative to establish whether adequate progress is being made towards the SDGs.
2017 UNHLPF on sustainable development (20)	43 countries from across the world	Compilation of Executive Summaries Voluntary National Reviews; 2017	Reporting countries have addressed the imperative of “leaving no one behind” in multiple ways. All reporting countries have included SDGs into national development plans and strategies and most have adopted robust institutional frameworks for guiding and monitoring the implementation of the 2030 Agenda. The challenges facing reporting countries in various areas are: collection, processing, analysis and dissemination of reliable, timely, accessible, and sufficiently disaggregated data. Awareness-raising among all stakeholders is crucial to accelerating implementation of the 2030 Agenda. Governments, civil society, business, and the wider public need to learn more about the SDGs to act upon them.
UNDG, 2017 (39)	Countries in the UN Latin America & the	Support of United Nations country teams to governments of Latin America and the Carribean for the	UN country teams in the region have supported each country in SDG implementation in various ways such as: raising public awareness; monitoring, reporting and accountability; reviewing existing strategies and plans at the national, sub-national and local level; support in institutional development such as inter-ministerial collaborations for multi-sectoral work; applying multi-stakeholder approaches; supporting policy coherence; and identifying financing and budgeting strategies to efficiently fund SDG implementation.

Author/Organisation, year	Setting	Title	Key findings related to implementation of health and health related sustainable development goals
	Carribean Region	implementation of the 2030 agenda.	
UNDESA, 2018 (26)	60 developed and developing countries	Working together: Integration, institutions and the sustainable development goals. World Public Sector Report 2018.	The documents outlines how three approaches to integrated implementation of SDGs i.e. vertical (across different government sectors), horizontal (across different levels of government from national to local), and engaging stakeholders is being implemented across the world.<br>The findings show that there are few examples of full and effective vertical integration across national, sub-national and local levels. For horizontal integration, overcoming sectoral boundaries is a challenge but many countries are making arrangements for such integration e.g. by instituting high-level commissions. In 60 countries studied, 17 had set-up new cross-sectoral entities for implementation of SDGs. In terms of stakeholder engagement, various stakeholders can bring various strengths and need to be engaged further. Health and well-being related integration is evident in many developed and developing countries through a health-in-all-policies approach e.g. Australia, Brazil, Cuba, Finland, Iran, Malaysia, New Zealand, Norway, Sri Lanka, Sweden, Thailand and the United Kingdom.
Lao, 2016 (36)	Lao	Report Round Table Implementation Meeting Lao People's Democratic Republic	The national Steering Committee for SDGs has been established. The National SDGs Secretariat will be established at the Ministry of Foreign Affairs. Development partners, civil society and private sector will continue to actively participate in the consultations around the SDGs and play an integral role in supporting their achievement at the national and sub-national levels. SDG focal nominated by line ministries, government agencies and provinces. In addition, each province will consider nominating SDG focal points at district- and village-level as appropriate. Key actions will be taken to strengthen the national statistical system and improve the quality and disaggregation of data. Vientiane Declaration and Action Plan highlighted with cross-collaborative approaches like round table conference enhancing multi-sectoral engagement.
Zambia, 2017, National assemble (42)	Zambia	Report Of the Committee on health, community development and social services. National assembly of Zambia.	Measures to achieve target 3.4 of health SDG include: Health promotion & education in communities, training of front line health workers, scaling up the de-addiction program for alcoholics and other drug addicts, providing guidelines for the management of NCDs at all levels of health care, Enforcement of legislation passed to control NCD and related risk factors; recruitment and deployment of human resources for health countrywide, construction and renovation of hospitals and health centres countrywide to manage NCDs, enhancing the referral system at all levels of health care. Funding from domestic, bilateral and multilateral channels should be explored including tax on health-harming products such as tobacco. Challenges are related to data collection system and standardization of data for monitoring.
UNICEF, 2017 (40)	Multiple countries across the world	UN Coherence in Action Delivering Results for Children.	UNICEF efforts in supporting countries implement health and related SDGs include: support in development of national plans and strategies, data ecosystem mapping, communication and advocacy change, supporting implementation for refugee and migrant communities and providing technical expertise.

Author/Organisation, year	Setting	Title	Key findings related to implementation of health and health related sustainable development goals
UNICEF, 2018 Guatemala, (28)	Guatemala	Joint Audit of the Sustainable Development Goals Fund Joint Programme in Guatemala	Based on the audit work performed, OIAI concluded at the end of the audit that governance, risk management and internal controls over were generally established and functioning during the period under audit.
